# Supplementary material for: Prognostic impact of the distance from the anterior surface to tumor cells in pancreatoduodenectomy with neoadjuvant chemoradiotherapy for pancreatic ductal adenocarcinoma
Source: PLoS One. 2024 Jul 26;19(7):e0307876. doi: 10.1371/journal.pone.0307876 (PMC11280245; doi:10.1371/journal.pone.0307876)
Supplement: Attachment — Submitted filename: PONE-D-24-08272_reviewed copy.pdf [file pone.0307876.s002.pdf]

PLOS ONE

Prognostic impact of the distance from the anterior surface to tumor cells in pancreatoduodenectomy with neoadjuvant chemoradiotherapy for pancreatic ductal adenocarcinoma

--Manuscript Draft--

Manuscript Number:	PONE-D-24-08272
Article Type:	Research Article
Full Title:	Prognostic impact of the distance from the anterior surface to tumor cells in pancreatoduodenectomy with neoadjuvant chemoradiotherapy for pancreatic ductal adenocarcinoma
Short Title:	Distance from the anterior surface to tumor cells and prognosis in PD with neoadjuvant therapy
Corresponding Author:	KATSUNORI UCHIDA<br>Kansai Medical University: Kansai Ika Daigaku<br>Tsu, MIE, Select region JAPAN
Keywords:	pancreatic ductal adenocarcinoma; pancreatoduodenectomy; neoadjuvant chemoradiotherapy
Abstract:	Purpose<br><br>Several reports have shown the importance of margins in pancreatoduodenectomy (PD) specimens; however, whether anterior surfaces are included as margins varies among reports. In this study, we aimed to examine the impact of the anterior surface on disease-free survival (DFS) and overall survival (OS).<br><br>Method<br><br>In total, 98 patients who underwent PD after chemoradiotherapy for pancreatic ductal adenocarcinoma at Mie University Hospital between January 1, 2012, and December 31, 2019, were included. We investigated the prognostic impact of the distance from the anterior surface to tumor cells on DFS and OS using the log-rank test. Multivariate analysis was performed using Cox proportional hazards analysis.<br><br>Results<br><br>A significant difference in DFS and OS was observed up to a distance of 5 mm from the anterior surface of tumor cells. Multivariate analysis revealed that the distance from the anterior surface to tumor cells ≤5 mm was an independent poor prognostic factor for DFS and OS.<br><br>Conclusion<br><br>In patients with PD treated with neoadjuvant therapy, the distance from the anterior surface to tumor cells is an important assessment and should be included in the pathology report.
Order of Authors:	Miki Usui
	Katsunori Uchida
	Aoi Hayasaki
	Masashi Kisiwada
	Shugo Mizuno
	Masatoshi Watanabe
Additional Information:	
**Question**	**Response**

Powered by Editorial Manager® and ProduXion Manager® from Aries Systems Corporation

**Financial Disclosure**<br><br>Enter a financial disclosure statement that describes the sources of funding for the work included in this submission. Review the submission guidelines for detailed requirements. View published research articles from *PLOS ONE* for specific examples.<br><br>This statement is required for submission and **will appear in the published article** if the submission is accepted. Please make sure it is accurate.<br><br>**Funded studies**<br>Enter a statement with the following details:<br>• Initials of the authors who received each award<br>• Grant numbers awarded to each author<br>• The full name of each funder<br>• URL of each funder website<br>• Did the sponsors or funders play any role in the study design, data collection and analysis, decision to publish, or preparation of the manuscript?<br><br>Did you receive funding for this work?	The author(s) received no specific funding for this work.
**Competing Interests**<br><br>Use the instructions below to enter a competing interest statement for this submission. On behalf of all authors, disclose any competing interests that could be perceived to bias this work—acknowledging all financial support and any other relevant financial or non-financial competing interests.<br><br>This statement is required for submission and **will appear in the published article** if the submission is accepted. Please make sure it is accurate and that any funding sources listed in your Funding Information later in the submission form are also declared in your Financial Disclosure statement.	The authors have declared that no competing interests exist.

Powered by Editorial Manager® and ProduXion Manager® from Aries Systems Corporation

View published research articles from *PLOS ONE* for specific examples.<br><br>**NO authors have competing interests**<br><br>Enter: *The authors have declared that no competing interests exist.*<br><br>**Authors with competing interests**<br><br>Enter competing interest details beginning with this statement:<br><br>*I have read the journal's policy and the authors of this manuscript have the following competing interests: [insert competing interests here]*<br><br>* typeset	
**Ethics Statement**<br><br>Enter an ethics statement for this submission. This statement is required if the study involved:<br><br>• Human participants<br>• Human specimens or tissue<br>• Vertebrate animals or cephalopods<br>• Vertebrate embryos or tissues<br>• Field research<br><br>Write "N/A" if the submission does not require an ethics statement.<br><br>General guidance is provided below. Consult the submission guidelines for detailed instructions. **Make sure that all information entered here is included in the Methods section of the manuscript.**	This study was approved by the Clinical Research Ethics Review Committee of Mie University Hospital, waiving the need for informed consent due to the unfeasibility of obtaining individual consent from many dead patients and the retrospective nature of the study (reference number: H2018-073). The patients were given the opportunity to opt out of the study at any time, with details available on the Mie University School of Medicine website, ensuring adherence to the ethical standards outlined in the Declaration of Helsinki.

Powered by Editorial Manager® and ProduXion Manager® from Aries Systems Corporation

**Format for specific study types**<br><br>**Human Subject Research (involving human participants and/or tissue)**<br>• Give the name of the institutional review board or ethics committee that approved the study<br>• Include the approval number and/or a statement indicating approval of this research<br>• Indicate the form of consent obtained (written/oral) or the reason that consent was not obtained (e.g. the data were analyzed anonymously)<br><br>**Animal Research (involving vertebrate animals, embryos or tissues)**<br>• Provide the name of the Institutional Animal Care and Use Committee (IACUC) or other relevant ethics board that reviewed the study protocol, and indicate whether they approved this research or granted a formal waiver of ethical approval<br>• Include an approval number if one was obtained<br>• If the study involved *non-human primates*, add *additional details* about animal welfare and steps taken to ameliorate suffering<br>• If anesthesia, euthanasia, or any kind of animal sacrifice is part of the study, include briefly which substances and/or methods were applied<br><br>**Field Research**<br><br>Include the following details if this study involves the collection of plant, animal, or other materials from a natural setting:<br>• Field permit number<br>• Name of the institution or relevant body that granted permission	
**Data Availability**<br><br>Authors are required to make all data underlying the findings described fully available, without restriction, and from the time of publication. PLOS allows rare exceptions to address legal and ethical concerns. See the PLOS Data Policy and FAQ for detailed information.	Yes - all data are fully available without restriction

A Data Availability Statement describing where the data can be found is required at submission. Your answers to this question constitute the Data Availability Statement and **will be published in the article**, if accepted.<br><br>**Important:** Stating 'data available on request from the author' is not sufficient. If your data are only available upon request, select 'No' for the first question and explain your exceptional situation in the text box.<br><br>Do the authors confirm that all data underlying the findings described in their manuscript are fully available without restriction?	
**Describe where the data may be found in full sentences. If you are copying our sample text, replace any instances of XXX with the appropriate details.**<br><br>• If the data are **held or will be held in a public repository**, include URLs, accession numbers or DOIs. If this information will only be available after acceptance, indicate this by ticking the box below. For example: *All XXX files are available from the XXX database (accession number(s) XXX, XXX.)*.<br>• If the data are all contained **within the manuscript and/or Supporting Information files**, enter the following: *All relevant data are within the manuscript and its Supporting Information files.*<br>• If neither of these applies but you are able to provide **details of access elsewhere**, with or without limitations, please do so. For example:<br><br>*Data cannot be shared publicly because of [XXX]. Data are available from the XXX Institutional Data Access / Ethics Committee (contact via XXX) for researchers who meet the criteria for access to confidential data.*<br><br>*The data underlying the results presented in the study are available from (include the name of the third party*	All relevant data are within the manuscript and its Supporting Information files.

and contact information or URL).<br>• This text is appropriate if the data are owned by a third party and authors do not have permission to share the data.<br><br>* typeset	
Additional data availability information:	Tick here if the URLs/accession numbers/DOIs will be available only after acceptance of the manuscript for publication so that we can ensure their inclusion before publication.

Powered by Editorial Manager® and ProduXion Manager® from Aries Systems Corporation

Manuscript Click here to access/download;Manuscript;Manuscript.docx

Prognostic impact of the distance from the anterior surface to tumor cells in pancreatoduodenectomy with neoadjuvant chemoradiotherapy for pancreatic ductal adenocarcinoma

Short title: Distance from the anterior surface to tumor cells and prognosis in PD with neoadjuvant therapy

Authors name

Miki Usui[1], Katsunori Uchida[1, 2*], Aoi Hayasaki[3], Masashi Kisiwada[3], Shugo Mizuno[3], Masatoshi Watanabe[1]

Affiliations

1. Department of Oncologic Pathology, Mie University Graduate School of Medicine, Tsu, Mie, Japan
2. Department of Pathology, Kansai Medical University, Hirakata, Osaka, Japan
3. Department of Hepato-Biliary-Pancreatic and Transplant Surgery, Mie University School of Medicine, Tsu, Mie, Japan

***Corresponding author** uchidkat@hirakata.kmu.ac.jp (KU)

E-mail: uchidkat@hirakata.kmu.ac.jp

Abstract

Purpose: Several reports have shown the importance of margins in pancreatoduodenectomy (PD) specimens; however, whether anterior surfaces are included as margins varies among reports. In this study, we aimed to examine the impact of the anterior surface on disease-free survival (DFS) and overall survival (OS).

Method: In total, 98 patients who underwent PD after chemoradiotherapy for pancreatic ductal adenocarcinoma at Mie University Hospital between January 1, 2012, and December 31, 2019, were included. We investigated the prognostic impact of the distance from the anterior surface to tumor cells on DFS and OS using a log-rank test. Multivariate analysis was performed using Cox proportional hazards analysis.

Results: A significant difference in DFS and OS was observed up to a distance of 5 mm from the anterior surface of tumor cells. The multivariate analysis revealed that the distance from the anterior surface to tumor cells (≤5 mm) was an independent poor prognostic factor for DFS and OS.

Conclusion: In patients with PD treated with neoadjuvant therapy, the distance from the anterior surface to tumor cells is an important assessment and should be included in the pathology report.

Introduction

Pancreatic ductal adenocarcinoma (PDAC) is a highly lethal disease, and the 5-year survival rate for all stages combined is 12%, according to the American Cancer Society [1]. Currently, the usefulness of neoadjuvant therapy has been reported not only for borderline disease but also for resectable disease [2]. The National Comprehensive Cancer Network (NCCN) guidelines indicate that neoadjuvant therapy is the first-line treatment for resectable or borderline resectable pancreatic cancer. Consequently, an increasing number of cases of chemoradiotherapy followed by surgical treatment is predicted in the future.

Several reports have examined prognostic factors in PD specimens, and the distance between the tumor cells and the margin is an independent prognostic factor [3–16]. Among these reports, some included

the anterior surface as a margin, whereas others did not, suggesting that it is debatable whether the anterior surface should be considered a margin. The College of American Pathologists (CAP) protocol specifies that the presence of tumor cells within 1 mm of the resection margin is considered a positive margin; however, this rule does not apply to the anterior and posterior surfaces because they are not resection margins. It also states that reporting tumor invasion on the anterior and non-uncinate posterior surfaces is recommended but not required [17]. One report showed that invasion of the anterior surface was a poor prognostic factor [3]. In contrast, another study reported that tumor cells less than 1 mm away from the anterior surface had no prognostic impact [4]. However, these reports were based on patients who did not receive neoadjuvant therapy, and the prognostic relationship between the margin and prognosis in patients receiving neoadjuvant therapy is unclear.

We investigated the prognostic impact of the anterior surface in PD specimens treated with neoadjuvant therapy.

Materials and methods

Patients

This study was approved by the Clinical Research Ethics Review Committee of Mie University Hospital, waiving the need for informed consent due to the unfeasibility of obtaining individual consent from many dead patients and the retrospective nature of the study (reference number: H2018-073). The patients were given the opportunity to opt out of the study at any time, with details available on the Mie University School of Medicine website, ensuring adherence to the ethical standards outlined in the Declaration of Helsinki. Patient clinical and pathological data were extracted from the hospital's electronic medical records, anonymized, and stored in a database, maintaining strict confidentiality and being used solely for research purposes. The data cutoff was June 23, 2023. We extracted the clinical and pathological data of 134 patients who underwent pancreatectomy following chemoradiotherapy with gemcitabine and S-1 for pancreatic ductal adenocarcinoma at Mie University Hospital from January 1, 2012, to December 31, 2019 (Fig 1). Among these patients, one underwent total pancreatectomy, and 24 underwent distal pancreatectomy. We excluded five cases with no residual

tumor, four cases where the tumor margin distance was indeterminable, and two cases of R2 resection.

Fig 1. Flow diagram of patient inclusion in the study.

Pathological examination

The portal vein (PV)/superior mesenteric vein (SMV) margin, superior mesenteric artery (SMA) margin, and posterior surface of the PD specimens were inked according to the color code assigned by the surgeon. The anterior area without inking was designated as the anterior surface area. The specimens were fixed overnight in a 10% neutral-buffered formalin solution. Pathologists sliced the PD specimen into 5-mm-thick slices in the axial plane, sampled all pancreatic tissues, and evaluated the pathological findings [18–20].

To validate the margins, the anterior surface and other margins (PV/SMV margin, SMA margin, and posterior surface) were validated as different factors. The distance from the anterior surface to the tumor cells and its impact on prognosis were examined at 0 mm, 0–X mm, and > X mm (X = 1, 2, 3, 4, 5, and 6). R1 was defined as the presence of tumor cells at a distance of 1 mm from any other margin except the anterior surface. Additionally, pancreatic transection, bile duct, and proximal/distal enteric margins were evaluated as independent factors.

Statistical analysis

The Kaplan–Meier method was used to analyze disease-free survival (DFS) and overall survival (OS), with the surgery date as the starting point. The log-rank test was used to compare survival time distributions between the groups. A multivariate analysis was performed using the Cox proportional hazards model to identify significant prognostic factors. Statistical significance was set at $p < 0.05$. All statistical analyses were performed using EZR (Saitama Medical Center, Jichi Medical University, Saitama, Japan), a graphical user interface for R (The R Foundation for Statistical Computing, Vienna, Austria) [21].

Results

Patient characteristics

Of the 98 patients, 57 were male and 41 were female. The mean age at diagnosis was 66.6 years and ranged from 41 to 86 years. Sixty-one (62.2%) patients died of pancreatic cancer, 18 (18.4%) died of other causes, and 19 (19.4%) survived. Seventy (71.4%) patients experienced recurrence. The median time to recurrence was 13.5 months, and the median survival time was 25.5 months. Eighty-seven (88.7%) patients underwent resection of the PV or SMV. The relationship between each clinicopathological factor and DFS/OS is presented in Table 1. In two patients, histological evaluation of extra-pancreatic nerve plexus invasion was impossible.

Table 1. Pathological and clinical findings and outcomes.

**Table 1. Pathological and clinical findings and outcomes**					
**variable**	**Number of patients (%)**	**Median DFS (months)**	**Log-rank p-value (DFS)**	**Median OS (months)**	**Log-rank p-value (OS)**
**Overall**	98	13.50		25.49	
**Age, years**					
**<65**	35 (35.7%)	16.43	0.156	32.56	0.104
**≧65**	63 (64.3%)	12.16		22.90	
**Gender**					
**Male**	57 (58.2%)	15.44	0.961	22.90	0.666
**Female**	41 (41.8%)	12.32		26.28	
**Performance status**					

**0**	68 (69.4%)	14.85	0.371	26.87	0.041
**1**	23 (23.5%)	12.68		22.87	
**2**	6 (6.1%)	8.16		14.34	
**3**	1 (1.0%)	8.25		9.86	
**Preoperative CA19-9**					
**≦37**	66 (68.0%)	21.91	0.048	29.47	0.278
**＞37**	31 (32.0%)	15.87		32.53	
**Resectability after neoadjuvant therapy**					
**R**	31 (31.6%)	29.80	0.057	43.04	0.034
**BR-PV**	19 (19.4%)	10.05		20.99	
**BR-A**	18 (18.4%)	11.14		24.87	
**UR-LA**	30 (30.6%)	11.24		19.55	
**Adjuvant therapy**					
**No adjuvant**	17 (17.3%)	7.72	0.092	7.79	0.017
**Adjuvant**	81 (82.7%)	14.26		29.50	
**Tumor size**					
**≦20**	35 (35.7%)	27.66	0.007	36.44	0.055
**>20**	63 (64.3%)	11.14		22.74	
**Tumor grade**					
**Well**	31 (31.6%)	20.99	0.467	26.87	0.689
**Moderate**	25 (25.5%)	9.86		21.39	
**Poor**	42 (42.9%)	13.04		29.50	
**Perineural invasion**					
**Negative**	38 (38.9%)	29.21	0.003	38.41	0.013
**Positive**	60 (61.2%)	11.99		22.74	

**Lymphovascular invasion**					
**Negative**	76 (77.6%)	16.49	0.035	26.87	0.234
**Positive**	22 (22.4%)	8.79		16.99	
**Extra-pancreatic nerve plexus invasion**					
**Negative**	63 (64.3%)	16.56	0.021	35.32	0.011
**Positive**	33 (35.7%)	11.83		22.77	
**Grading of histological response [a]**					
**1**	28 (28.6%)	15.21	0.037	35.91	0.032
**2**	65 (66.3%)	11.83		21.78	
**3**	5 (5.1%)	29.80		34.60	
**pT-stage [b]**					
**1**	39 (39.8%)	27.86	0.0011	41.43	0.054
**2**	51 (52.0%)	10.05		22.21	
**3**	7 (7.1%)	4.57		12.71	
**4**	1 (1.0%)	19.81		32.30	
**pN-stage [b]**					
**0**	67 (68.4%)	15.44	0.603	26.87	0.717
**1**	21 (21.4%)	12.94		25.82	
**2**	10 (10.2%)	4.22		12.63	
**Margin status**					
**R0 (>1 mm)**	64 (65.3%)	15.93	0.105	26.58	0.371
**R1 (≦1 mm)**	34 (34.7%)	12.07		22.90	

Abbreviation: DFS: disease-free survival; DSS: disease-specific survival; OS: overall survival; R: resectable;

BR-PV: borderline resectable-portal vein; BR-A: borderline resectable-artery; UR-LA: unresectable-locally advanced.

a College of American Pathologists system.

b Union for International Cancer Control (UICC) TNM Classification of Malignant Tumors (ed 8), 2018.

Impact of the distance from the margin to the tumor cells

Log-rank tests showed a statistically significant difference between groups with a distance from the anterior surface to the tumor cells of X mm or less and greater than X mm for X = 1, 2, 3, 4, and 5. However, there was a significant difference in DFS ($p = 0.027$) but not in OS ($p = 0.059$) at X=6 (Figs 2 and 3); when $X \leq 5$ mm, significant differences in DFS and OS were shown between the groups. In contrast, margins other than the anterior surface (R1) were not prognostic for DFS or OS ($p > 0.05$) (Table 1). No tumor cells were within 1 mm of the pancreatic transection, bile duct, or proximal/distal enteric margins. Multivariate analysis also showed that tumor cells within X mm of the anterior surface were an independent poor prognostic factor for DFS and OS at X = 1, 2, 3, 4, and 5 (Table 2).

Fig 2. Kaplan–Meier curves for 98 patients stratified into two groups by the distance from the anterior surface to tumor cells for DFS.

Fig 3. Kaplan–Meier curves for 98 patients stratified into two groups by the distance from the anterior surface to tumor cells for OS.

Table 2. Multivariate analysis.

Table 2. Multivariate analysis.

Variable	DFS			OS		
	HR	95% CI	p-value	HR	95% CI	p-value
**0 mm**						
**Resectability after neoadjuvant therapy, BR-PV+BR-A+UR-LA**	1.535	0.904–2.608	0.113	1.576	0.922–2.693	0.097
**Tumor size >20 mm**	1.305	0.747–2.280	0.349	1.116	0.628–1.983	0.709
**Perineural invasion, positive**	1.496	0.816–2.745	0.193	1.389	0.756–2.554	0.290
**Lymphovascular invasion, positive**	1.551	0.899–2.676	0.115	1.366	0.794–2.351	0.260
**Extra-pancreatic nerve plexus invasion, positive**	1.004	0.579–1.741	0.989	1.163	0.665–2.033	0.598
**Grading of histological response, Score 3**	1.626	0.917–2.885	0.096	1.848	1.039–3.286	0.037
**Distance from the anterior surface to the tumor cells: 0 mm**	8.761	1.884–40.740	0.006	31.70	5.886–170.800	p<0.001
**1.0 mm**						
**Resectability after neoadjuvant therapy, BR-PV+BR-A+UR-LA**	1.419	0.825–2.439	0.206	1.481	0.856–2.561	0.160
**Tumor size >20 mm**	1.331	0.766–2.315	0.311	1.179	0.666–2.088	0.572
**Perineural invasion, positive**	1.495	0.810–2.758	0.199	1.342	0.728–2.472	0.346

**Lymphovascular invasion, positive**	1.720	0.985–3.002	0.056	1.289	0.750–2.216	0.358
**Extra-pancreatic nerve plexus invasion, positive**	0.844	0.475–1.502	0.565	0.941	0.520–1.702	0.841
**Grading of histological response, Score 3**	1.496	0.847–2.643	0.165	1.765	1.002–3.110	0.049
**Distance from the anterior surface to the tumor cells: ≤1 mm**	2.765	1.422–5.379	0.003	2.966	1.512–5.820	0.002
**2.0 mm**						
**Resectability after neoadjuvant therapy, BR-PV+BR-A+UR-LA**	1.480	0.870–2.516	0.148	1.492	0.870–2.559	0.147
**Tumor size, >20 mm**	1.213	0.689–2.134	0.503	1.056	0.591–1.889	0.854
**Perineural invasion, positive**	1.516	0.823–2.791	0.182	1.438	0.783–2.639	0.241
**Lymphovascular invasion, positive**	1.758	1.015–3.047	0.044	1.410	0.822–2.421	0.212
**Extra-pancreatic nerve plexus invasion, positive**	0.751	0.415–1.359	0.344	0.828	0.448–1.530	0.547
**Grading of histological response, Score 3**	1.407	0.793–2.498	0.243	1.605	0.908–2.840	0.104
**Distance from the anterior surface to the tumor cells: ≤2 mm**	2.880	1.585–5.234	p<0.001	3.078	1.631–5.809	p<0.001
**3.0 mm**						

**Resectability after neoadjuvant therapy, BR-PV+BR-A+UR-LA**	2.225	1.277–3.875	0.005	2.171	1.245–3.786	0.006
**Tumor size, >20 mm**	1.220	0.699–2.131	0.484	1.070	0.602–1.902	0.817
**Perineural invasion, positive**	1.236	0.670–2.283	0.498	1.132	0.617–2.079	0.689
**Lymphovascular invasion, positive**	1.654	0.963–2.839	0.068	1.367	0.799–2.338	0.253
**Extra-pancreatic nerve plexus invasion, positive**	0.794	0.446–1.412	0.432	0.948	0.533–1.686	0.855
**Grading of histological response, Score 3**	1.563	0.877–2.785	0.130	1.704	0.961–3.024	0.068
**Distance from the anterior surface to the tumor cells: ≤3 mm**	2.650	1.561–4.497	p<0.001	2.468	1.452–4.195	p<0.001
**4.0 mm**						
**Resectability after neoadjuvant therapy, BR-PV+BR-A+UR-LA**	2.152	1.251–3.700	0.006	2.113	1.226–3.640	0.007
**Tumor size, >20 mm**	1.072	0.613–1.875	0.806	0.944	0.530–1.683	0.845
**Perineural invasion, positive**	1.579	0.871–2.861	0.132	1.349	0.750–2.428	0.318
**Lymphovascular invasion, positive**	1.845	1.063–3.202	0.029	1.493	0.867–2.572	0.148
**Extra-pancreatic nerve plexus invasion, positive**	0.804	0.459–1.406	0.443	0.981	0.561–1.713	0.945

**Grading of histological response, Score 3**	1.455	0.826–2.566	0.195	1.676	0.952–2.950	0.074
**Distance from the anterior surface to the tumor cells, ≤4 mm**	2.684	1.605–4.487	p<0.001	2.498	1.496–4.169	p<0.001
**5.0 mm**						
**Resectability after neoadjuvant therapy, BR-PV+BR-A+UR-LA**	1.735	1.034–2.912	0.037	1.716	1.018–2.892	0.043
**Tumor size, >20 mm**	1.149	0.650–2.032	0.634	1.022	0.568–1.840	0.942
**Perineural invasion, positive**	1.448	0.795–2.637	0.226	1.291	0.710–2.348	0.403
**Lymphovascular invasion, positive**	1.806	1.031–3.163	0.039	1.493	0.860–2.593	0.155
**Extra-pancreatic nerve plexus invasion, positive**	0.991	0.577–1.701	0.973	1.167	0.675–2.021	0.580
**Grading of histological response, Score 3**	1.398	0.793–2.463	0.247	1.598	0.908–2.812	0.104
**Distance from the anterior surface to the tumor cells ≤5 mm**	1.964	1.211–3.187	0.006	1.789	1.105–2.895	0.018

Abbreviation: HR: hazard ratio; CI: confidence intervals.

Discussion

Several studies have examined the relationship between the distance from the margin to tumor cells and the prognosis of PD specimens. However, there are limited data on the prognostic impact of the distance between the anterior surface and tumor cells and the validation of PD specimens treated with

neoadjuvant therapy. We showed that tumor cells within 5 mm of the anterior surface were associated with a poorer OS prognosis than those in the >5 mm group. In contrast, tumor cells within 1 mm of the margin, excluding the anterior surface (R1), did not affect DFS or OS.

Several reports have examined the distance between the margin and tumor cells and the prognosis in PD specimens without neoadjuvant therapy [3–16]. Some of these reports have not validated the anterior surfaces, likely because they were not resection margins [5,6]. In addition, some reports did not describe the margins subjected to validation in detail [7,8]. In these reports, depending on the study, the range of distances from the margin to the tumor cells that affected DFS and OS was from 0 to 2 mm. This may be caused by the differences between studies in the targeted margins, the distance defined as "a positive margin," whether or not inking was used, the extent of sampling, and so on. Few studies have examined individual margins. Regarding the distance between the anterior surface and tumor cells, one report showed that invasion of the anterior surface is a poor prognostic factor for 3-year survival [3], whereas another report showed that the presence of tumor cells within 1 mm of the anterior surface is associated with poor DFS [9]. However, some reports have shown that tumor cells within 0–2 mm of the anterior surface did not affect OS [4,10]. All these reports were based on patients who did not receive neoadjuvant therapy. In our study, the distance from the anterior surface to the tumor cells for better DFS and OS was longer than that in previous reports. This could have been caused by neoadjuvant therapy; however, this could not be clarified. Few studies have investigated the relationship between the margins and prognosis of PD specimens after neoadjuvant therapy. Although one report indicated that the presence of tumor cells within 1 mm of the margin was associated with poor DFS and OS, no details were provided regarding which margins were validated [7]. Another report showed that the presence of a tumor within 1 mm of any of the margins was associated with poor DFS and OS, targeting the superior mesenteric/medial, pancreatic neck, bile duct, proximal gastric or duodenal, and distal jejunal margins, anterior surface, and posterior surface, but this was not verified for each margin [11].

Several reports have been published on the prognostic impact of tumor cell distance from the margins in PD specimens. However, no definite conclusion has been reached because of the varying verification methods used in individual reports. The prognostic impact of the distance from an individual margin to

tumor cells depends on the margin to which the tumor cells are in proximity [3,11,13]; further studies using appropriate inking and sampling specimens are required.

Limitation

Our study has some limitations. First, this was a retrospective study and did not correct for patient characteristics. Second, this study was performed at a single center, and the influence of random errors caused by a relatively small number of cases could not be ruled out. Therefore, a multicenter study with a larger number of patients is required.

Conclusion

This study is the first to focus on the anterior surface as the distance from the tumor margin in PD specimens treated with neoadjuvant therapy. We showed that tumor cells within 5 mm of the anterior surface were a poor prognostic factor for recurrence and survival. Therefore, the pathological report should describe the distance from the anterior surface to the tumor cells.

Acknowledgments

The authors thank the members of the Departments of Oncological Pathology and Hepato-Biliary-Pancreatic and Transplant Surgery at Mie University for their valuable discussions and support.

References

1. American Cancer Society; 2023 [Cited January 14, 2024]. In: Cancer Statistics Center [Internet]. Available from: cancerstatisticscenter.cancer.org.

2. Motoi F, Kosuge T, Ueno H, Yamaue H, Satoi S, Sho M, et al. Randomized phase II/III trial of

neoadjuvant chemotherapy with gemcitabine and S-1 versus upfront surgery for resectable pancreatic cancer (Prep-02/JSAP05). Jpn J Clin Oncol. 2019;49: 190–194. doi:10.1093/jjco/hyy190

3. Nagakawa T, Sanada H, Inagaki M, Sugama J, Ueno K, Konishi I, et al. Long-term survivors after resection of carcinoma of the head of the pancreas: significance of histologically curative resection. J Hepatobiliary Pancreat Surg. 2004;11: 402–408. doi:10.1007/s00534-004-0917-4

4. Campbell F, Smith RA, Whelan P, Sutton R, Raraty M, Neoptolemos JP, et al. Classification of R1 resections for pancreatic cancer: the prognostic relevance of tumour involvement within 1 mm of a resection margin. Histopathology. 2009;55: 277–283. doi:10.1111/j.1365-2559.2009.03376.x

5. Obonyo D, Uslar VN, Münding J, Weyhe D, Tannapfel A. The impact of resection margin distance on survival and recurrence in pancreatic ductal adenocarcinoma in a retrospective cohort analysis. PLoS One. 2023;18: e0281921. doi:10.1371/journal.pone.0281921

6. Chang DK, Johns AL, Merrett ND, Gill AJ, Colvin EK, Scarlett CJ, et al. Margin clearance and outcome in resected pancreatic cancer. J Clin Oncol. 2009;27: 2855–2862. doi:10.1200/jco.2008.20.5104

7. Chopra A, Zenati M, Hogg ME, Zeh HJ III, Bartlett DL, Bahary N, et al. Impact of neoadjuvant therapy on survival following margin-positive resection for pancreatic cancer. Ann Surg Oncol. 2021;28: 7759–7769. doi:10.1245/s10434-021-10175-y

8. Ocuin LM, Miller-Ocuin JL, Zenati MS, Vargo JA, Singhi AD, Burton SA, et al. A margin distance analysis of the impact of adjuvant chemoradiation on survival after pancreatoduodenectomy for pancreatic adenocarcinoma. J Gastrointest Oncol. 2017;8: 696–704. doi:10.21037/jgo.2017.04.02

9. Sohn HJ, Kim H, Kim SJ, Lee KB, Han Y, Lee JM, et al. Oncologic outcomes according to the location and status of resection margin in pancreas head cancer: role of radiation therapy in R1 resection.

Ann Surg Treat Res. 2022;102: 10. doi:10.4174/astr.2022.102.1.10

10. Osipov A, Nissen N, Rutgers J, Dhall D, Naziri J, Chopra S, et al. Redefining the positive margin in pancreatic cancer: Impact on patterns of failure, long-term survival and adjuvant therapy. Ann Surg Oncol. 2017;24: 3674–3682. doi:10.1245/s10434-017-6076-z

11. Takahashi C, Shridhar R, Huston J, Meredith K. Adjuvant therapy for margin positive pancreatic cancer: A propensity score matched analysis. Pancreatology. 2022;22: 396–400. doi:10.1016/j.pan.2022.03.008

12. Kaltenmeier C, Nassour I, Hoehn RS, Khan S, Althans A, Geller DA, et al. Impact of resection margin status in patients with pancreatic cancer: A national cohort study. J Gastrointest Surg. 2021;25: 2307–2316. doi:10.1007/s11605-020-04870-6

13. Jamieson NB, Chan NIJ, Foulis AK, Dickson EJ, McKay CJ, Carter CR. The prognostic influence of resection margin clearance following pancreaticoduodenectomy for pancreatic ductal adenocarcinoma. J Gastrointest Surg. 2013;17: 511–521. doi:10.1007/s11605-012-2131-z

14. Janot MS, Kersting S, Belyaev O, Matuschek A, Chromik AM, Suelberg D, et al. Can the new RCP R0/R1 classification predict the clinical outcome in ductal adenocarcinoma of the pancreatic head? Langenbecks Arch Surg. 2012;397: 917–925. doi:10.1007/s00423-012-0953-5

15. Jamiyan T, Shiraki T, Kurata Y, Ichinose M, Kubota K, Imai Y. Clinical impacts of resection margin status and clinicopathologic parameters on pancreatic ductal adenocarcinoma. World J Surg Oncol. 2020;18. doi:10.1186/s12957-020-01900-0

16. Maeda S, Moore AM, Yohanathan L, Hata T, Truty MJ, Smoot RL, et al. Impact of resection margin status on survival in pancreatic cancer patients after neoadjuvant treatment and

pancreatoduodenectomy. Surgery. 2020;167: 803–811. doi:10.1016/j.surg.2019.12.008

17. The College of American Pathologists. Cancer protocol templates. 2023 [Cited January 14, 2024]. In: Protocols and templates [Internet]. Available from: https://www.cap.org/protocols-and-guidelines/cancer-reporting-tools/cancer-protocol-templates.

18. Verbeke CS. Resection margins and R1 rates in pancreatic cancer – are we there yet? Histopathology. 2008;52: 787–796. doi:10.1111/j.1365-2559.2007.02935.x

19. Verbeke CS, Leitch D, Menon KV, McMahon MJ, Guillou PJ, Anthoney A. Redefining the R1 resection in pancreatic cancer. Br J Surg. 2006;93: 1232–1237. doi:10.1002/bjs.5397

20. Adsay NV, Basturk O, Saka B, Bagci P, Ozdemir D, Balci S, et al. Whipple made simple for surgical pathologists: Orientation, dissection, and sampling of pancreaticoduodenectomy specimens for a more practical and accurate evaluation of pancreatic, distal common bile duct, and ampullary tumors. Am J Surg Pathol. 2014;38: 480–493. doi:10.1097/pas.0000000000000165

21. Kanda Y. Investigation of the freely available easy-to-use software ‘EZR’ for medical statistics. Bone Marrow Transplant. 2013;48: 452–458. doi:10.1038/bmt.2012.244

Supporting information

S1 Table. Patient data used for this study.

Figure
Click here to access/download;Figure;Fig1.tif

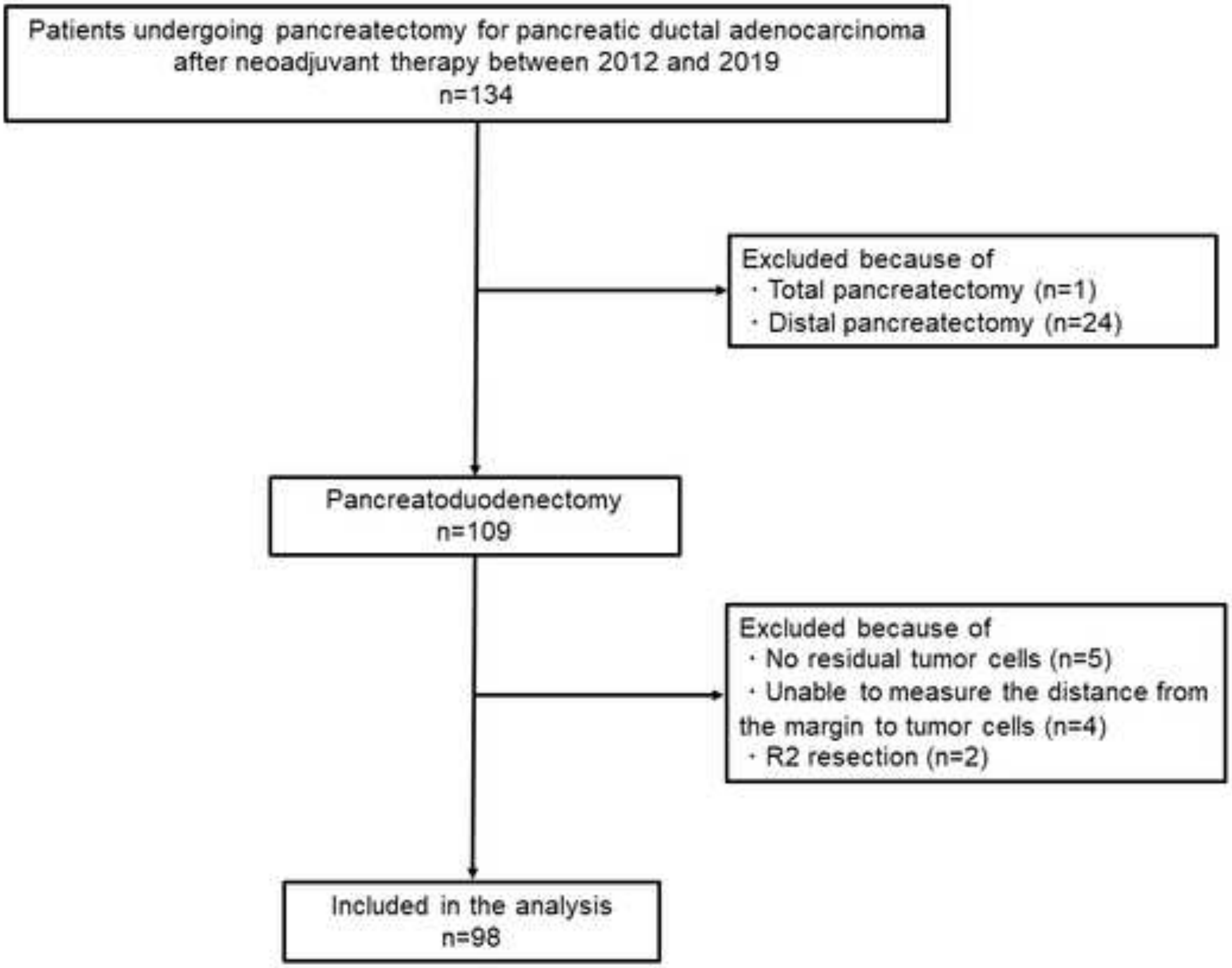

Fig 1. Flow diagram of patient inclusion in the study.

Figure

Click here to access/download;Figure;Fig2.tif

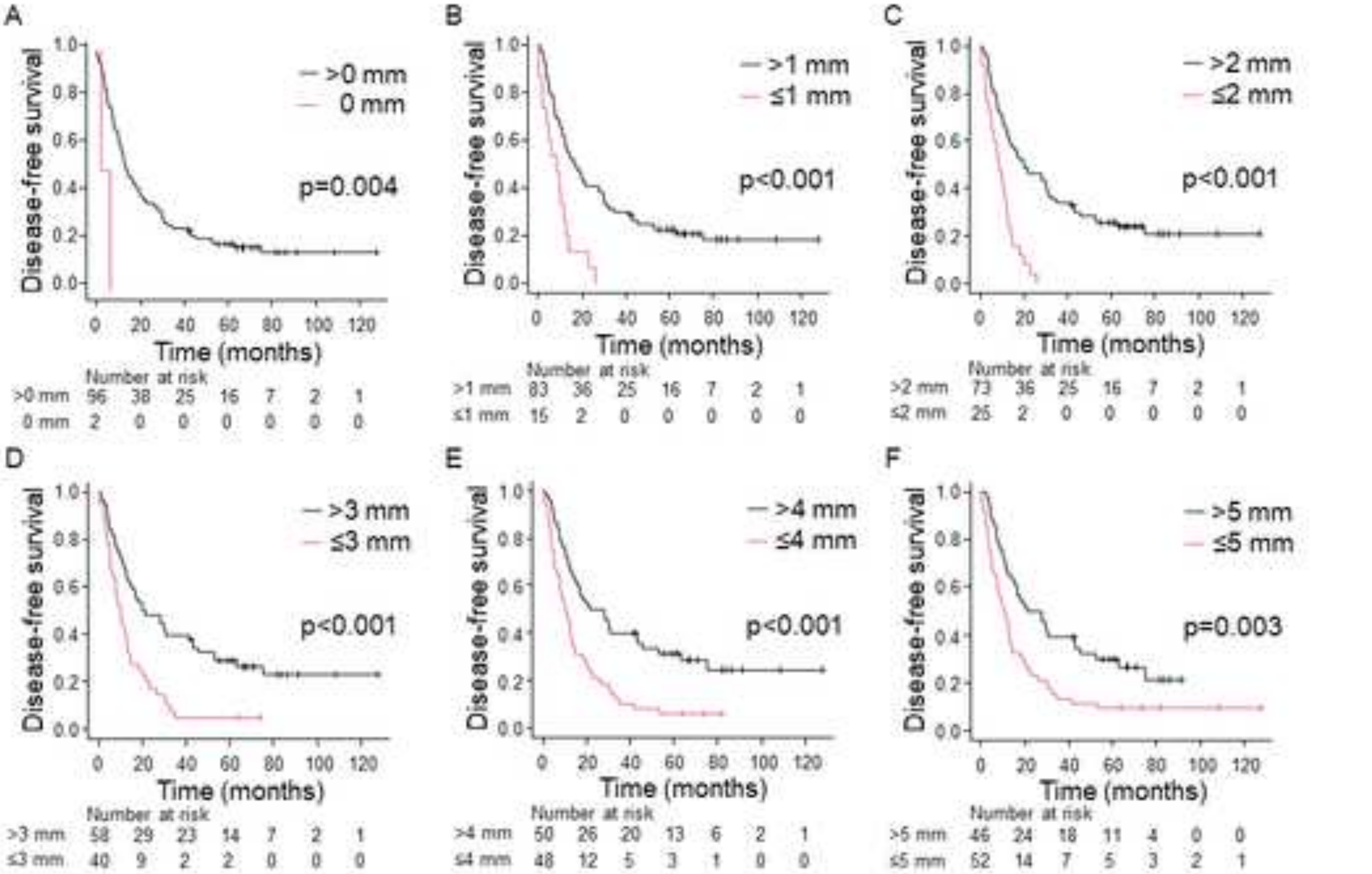

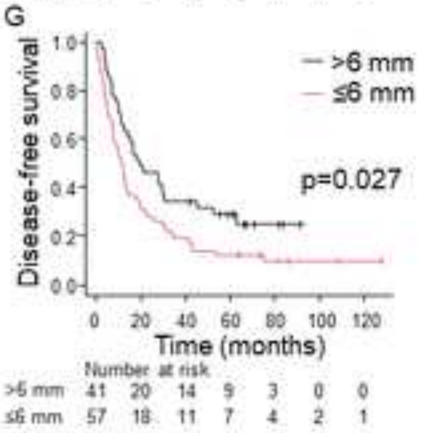

Fig 2. Kaplan-Meier curves for 98 patients stratified into two groups by the distance from anterior surface to tumor cells for DFS.
(A) 0 mm, (B) 1 mm, (C) 2 mm, (D) 3 mm, (E) 4 mm, (F) 5mm, (G) 6 mm

 Click here to access/download;Figure;Fig3.tif

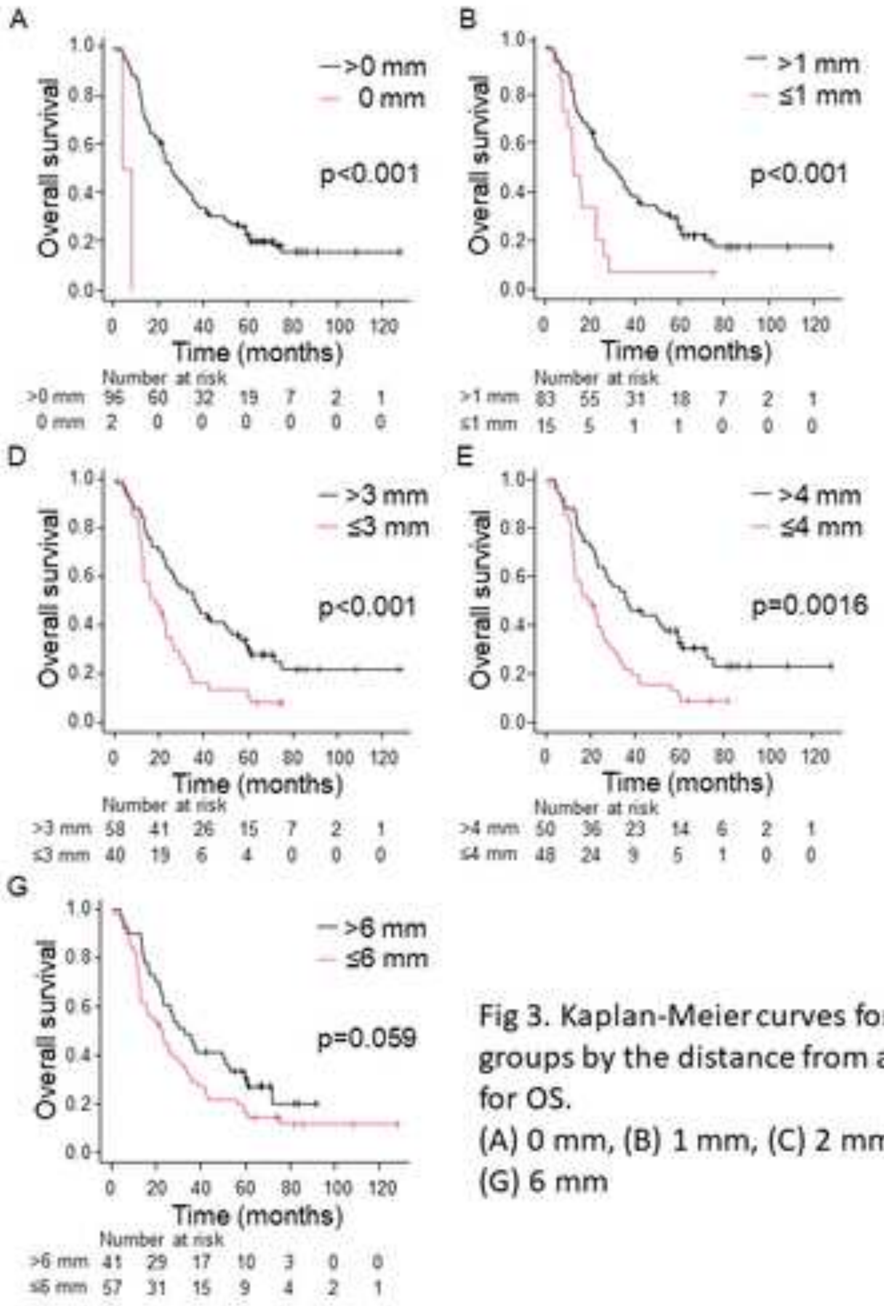

Fig 3. Kaplan-Meier curves for 98 patients stratified into two groups by the distance from anterior surface to tumor cells for OS.
(A) 0 mm, (B) 1 mm, (C) 2 mm, (D) 3 mm, (E) 4 mm, (F) 5mm, (G) 6 mm

Click here to access/download

Supporting Information

S1 Table.xlsx
