# Supplementary material for: Prognostic impact of the distance from the anterior surface to tumor cells in pancreatoduodenectomy with neoadjuvant chemoradiotherapy for pancreatic ductal adenocarcinoma
Source: PLoS One. 2024 Jul 26;19(7):e0307876. doi: 10.1371/journal.pone.0307876 (PMC11280245; doi:10.1371/journal.pone.0307876)
Supplement: Attachment — Submitted filename: Response to Reviewes.docx [file pone.0307876.s003.docx]

July 7th 2024

Paolo Aurello

Academic Editor

*PLOS ONE*

Response to Reviewer #1:

Thank you very much for reviewing our manuscript and offering valuable suggestions.

We have addressed your comments with point-by-point responses and revised the manuscript accordingly.

Comments 1 and 2: The authors do not provide a clear explanation of the biological reasoning behind their conclusions, and the challenges of assessing post-NAT specimens, however, they do mention that "In our study, the distance from the anterior surface to the tumor cells for better DFS and OS was longer than that in previous reports. This could have been caused by neoadjuvant therapy; however, this could not be clarified" [1]

The authors should cite and consider recent publications on the prognostic value of margin clearance [2]

Response: We appreciate the helpful suggestion. Following the reviewer's comment, we have compared our conclusions with those of recent reports on the prognostic value of margin clearance. Many of these reports did not clearly describe the procedure for sampling, and, likely, the search was not adequate. We have added the following to the Discussion (p.13, lines 144-151 and p13, lines 157-p14, lines 170):

Regarding the longer distance from the anterior surface to tumor cells required to improve DFS and OS than previously reported, we have highlighted the method of tissue sampling (p.13, lines 144-151) and discrepancies in diagnosis between pathologists (p13, lines 157-p14, lines 170).

“This discrepancy may be due to neither of these reports providing a detailed description of their tissue sampling methods and potentially sampling less tissue compared to our method, which sampled all pancreatic tissue. In pancreatectomy specimens post-preoperative adjuvant therapy, validated studies with extensively sampled specimens showed a pathological complete response rate of 2.5% [22], whereas validated studies with fewer sampled specimens tended to report higher rates [23–24]. This suggests that a greater number of samples increases the detection rate of tumor cells, which may also impact the assessment of the distance from margins to the tumor cells.”

“A report analyzing the prognostic significance of tumor cell distance from margins in total and distal pancreatectomy following neoadjuvant therapy revealed that in total pancreatectomy, the proximity of tumor cells to the SMA margin impacts OS, whereas the distance from other margins does not influence survival. In contrast, for distal pancreatectomy, the proximity of tumor cells to both the anterior and posterior surfaces does not affect survival [25]. Extensive tissue sampling was conducted for pathological examinations in this study. Patients undergoing either total or distal pancreatectomy generally present with fewer symptoms of biliary stenosis and typically have more advanced tumors at diagnosis compared to those undergoing pancreatoduodenectomy; therefore, our findings are not directly comparable. Moreover, in PDAC, the closeness of tumor cells to the anterior surface is not the sole determinant of OS. Furthermore, distinguishing between non-neoplastic ductal cells and intraepithelial and invasive carcinoma in pancreatectomy specimens after preoperative adjuvant therapy can be challenging. Accurate pathological assessment is essential, as diagnostic discrepancies among pathologists may influence study outcomes [26].”

Response to Reviewer #2:

Thank you very much for reviewing our manuscript and offering valuable suggestions.

We have addressed your comments with point-by-point responses and revised the manuscript accordingly.

Comment: A patient data is missing is very important for the practice of general surgery. The manuscript is well written. The data used are secondary and the institutional ethical approval is appropriate. Exclusion criteria are well stated. There is a patient data missing in the CA-19-9 result. Please cross-check.

Response: We appreciate the helpful suggestion. Following the reviewer's comment, we can confirm that preoperative serum CA19-9 levels had not been measured in one patient; we have changed the following text in the Result (p.5, lines 103-105)

Histological evaluation of extra-pancreatic nerve plexus invasion was impossible in two patients.

In one patient, preoperative serum CA19-9 levels were not measured, and in two patients, histological evaluation of extra-pancreatic nerve plexus invasion was impossible.

Thank you again for your comments on our manuscript. I trust that the revised manuscript now meets the standards for publication in *PLOS ONE*.

Sincerely,

Katsunori Uchida

Department of Pathology, Kansai Medical University, Osaka, Japan

2-3-1 Shinmachi, Hirakata, Osaka, 573-1191, Japan

Phone number: +81-72-804-0101

Fax number: +81-72-804-2960

Email address: uchidkat@hirakata.kmu.ac.jp
